# Laboratory-Based Examination of the Reliability and Validity of Kinematic Measures of Wrist and Finger Function Collected by a Telerehabilitation System in Persons with Chronic Stroke

**DOI:** 10.3390/s23052656

**Published:** 2023-02-28

**Authors:** Ashley MontJohnson, Amanda Cronce, Qinyin Qiu, Jigna Patel, Mee Eriksson, Alma Merians, Sergei Adamovich, Gerard Fluet

**Affiliations:** 1 Department of Biomedical Engineering, New Jersey Institute of Technology, Newark, NJ 07105, USA; 2 NeurotechR3 Inc., Warren, NJ 07059, USA; 3 School of Health Professions, Department of Rehabilitation and Movement Sciences, Rutgers, The State University of New Jersey, Newark, NJ 07102, USA

**Keywords:** stroke, rehabilitation, kinematics, wrist, hand, fingers

## Abstract

We have developed the New Jersey Institute of Technology—Home Virtual Rehabilitation System (NJIT—HoVRS) to facilitate intensive, hand-focused rehabilitation in the home. We developed testing simulations with the goal of providing richer information for clinicians performing remote assessments. This paper presents the results of reliability testing examining differences between in-person and remote testing as well as discriminatory and convergent validity testing of a battery of six kinematic measures collected with NJIT—HoVRS. Two different groups of persons with upper extremity impairments due to chronic stroke participated in two separate experiments. Data Collection: All data collection sessions included six kinematic tests collected with the Leap Motion Controller. Measurements collected include hand opening range, wrist extension range, pronation-supination range, hand opening accuracy, wrist extension accuracy, and pronation-supination accuracy. The system usability was evaluated by therapists performing the reliability study using the System Usability Scale. When comparing the in-laboratory collection and the first remote collection, the intra-class correlation coefficients (ICC) for three of the six measurements were above 0.900 and the other three were between 0.500 and 0.900. Two of the first remote collection/second remote collection ICCs were above 0.900, and the other four were between 0.600 and 0.900. The 95% confidence intervals for these ICC were broad, suggesting that these preliminary analyses need to be confirmed by studies with larger samples. The therapist’s SUS scores ranged from 70 to 90. The mean was 83.1 (SD = 6.4), which is consistent with industry adoption. There were statistically significant differences in the kinematic scores when comparing unimpaired and impaired UE for all six measures. Five of six impaired hand kinematic scores and five of six impaired/unimpaired hand difference scores demonstrated correlations between 0.400 and 0.700 with UEFMA scores. Reliability for all measures was acceptable for clinical practice. Discriminant and convergent validity testing suggest that scores on these tests may be meaningful and valid. Further testing in a remote setting is necessary to validate this process.

## 1. Introduction

Cerebrovascular accident (CVA) or stroke, is a leading cause of long-term disability in adults [1]. Despite more than a decade of investigating innovative approaches to rehabilitation, many individuals are left with upper limb deficits that hinder their ability to function independently [2]. Persistent, hand-related disability has a substantial effect on the productivity of the growing cohort of younger persons with milder strokes [3] and increases the cost of care for older and more impaired persons with strokes [4]. Current service delivery models emphasizing short, independence focused in-patient rehabilitation stays [5] and intermittent, low volume outpatient rehabilitation sessions [6] restrict the amount of rehabilitation a patient receives. This points to the need for rehabilitation services that patients can perform independently at home.

Recently, innovative telerehabilitation systems have been developed using information and communication technologies to provide rehabilitation services at a distance. A 2020 Cochrane Review of telerehabilitation interventions focusing on upper extremity function in persons with stroke demonstrated that persons receiving telerehabilitation made similar improvements in motor function as compared to in-person interventions [7]. We have developed the New Jersey Institute of Technology—Home Virtual Rehabilitation System (NJIT—HoVRS) to facilitate intensive, arm, wrist, hand, and finger-focused rehabilitation in the home [8]. The system integrates a Leap Motion Controller^®^ (LMC) and a suite of custom designed simulations to provide rehabilitation of the upper extremity through engaging games. Preliminary studies of NJIT—HoVRS support that persons with stroke can use this system effectively in the home with minimal remote supervision [8] and that performing a 12-week program of hand and arm training results in meaningful improvements in hand and arm function [9,10].

A smaller body of literature exists examining the measurement of movement in a telerehabilitation setting. Small studies of telerehabilitation-based outcome measures suggest that shoulder and elbow motor function of persons with stroke can be measured meaningfully by performing standard clinical tests using camera-based approaches. These measurements are limited to performance-based tests and measurements of range of motion [11]. Similar to in-person testing, remotely collected measures that focus purely on range of motion or task outcomes fail to differentiate between recovery processes and the development of efficient compensatory strategies [12]. Several authors cite kinematic analysis as a means to identify the normalization of motor function in persons with stroke [12,13,14]. Therefore, reliable and valid kinematic measurements that can be collected at home may allow therapists to adjust plans of care in a manner that would emphasize the normalization of movement patterns, possibly making interventions prescribed based on these tests more effective [15]. To address this gap, we designed a set of kinematic measures of wrist and finger movement that can be conducted remotely in an attempt to add to the ability of clinicians to measure motor recovery as it occurred in persons using NJIT—HoVRS in a telerehabilitation environment [8].

Kinematic measures captured by NJIT—HoVRS are collected via the LMC, which utilizes a pair of cameras and a set of infrared light emitting diodes (LEDs). Images collected by the cameras are transmitted via USB to the LMC’s tracking software, which analyzes and transforms the images into three dimensional representations of these images. This allows for real time, camera-based estimation of wrist and finger angles and position measures without an expensive and cumbersome wearable apparatus. Published psychometrics of LMC based position and movement measurements have been mixed, with static measures generally performing better than dynamic measures [16,17,18]. Studies of the LMC with a more clinical focus demonstrate that joint specific wrist and finger position and range of motion data collected by the LMC correlated with measurements collected with an electro-goniometer in a population of healthy individuals in their 20s [19]. In another study, repetitive wrist and finger movements produced changes in position data that correlated well with impairment level in persons with Parkinson’s disease [20].

A study by Gieser et al. utilized finger positions and joint angles to classify hand gestures in children with cerebral palsy [21]. Taken together, these studies begin to suggest that the LMC produces measurements that may be sufficiently accurate for clinical purposes.

We developed a set of testing simulations to accompany NJIT—HoVRS’ intervention simulations to collect a battery of kinematic measures with the goal of providing richer information for clinicians performing remote assessments of their stroke patients. As a first step toward validating these kinematic measurements remotely at home, this paper will describe the results of two different studies examining: (1) reliability testing focused on the agreement between in laboratory and remotely collected measurements as well as a test of the system’s usability in the remote collection mode; and (2) discriminatory as well as convergent validity testing of a battery of six kinematic measures collected with the NJIT—HoVRS system in a laboratory setting.

## 2. Study #1

### 2.1. Materials and Methods

All studies were approved by the institutional review boards of Rutgers, the State University of New Jersey, and the New Jersey Institute of Technology. 

#### 2.1.1. Subjects

Persons with Stroke: Subjects were recruited through stroke support group activities and two assistive technology fairs. Inclusion: (1) persons at least six months post stroke, (2) persons that were between 20 and 90 years of age; and (3) pre-training upper extremity Fugl-Meyer (UEFMA) scores between 20 and 56 [22]. Exclusion: (1) visual impairments making it impossible to interact with a 24-inch computer monitor, which was confirmed using the training system; (2) proprioceptive impairments making it impossible for subjects to move their hand reliably without looking at it, which was confirmed using the training system; (3) cognitive impairments that precluded the ability to participate in informed consent (Montreal Cognitive Assessment score less than 18 [23]). All subjects were free from neurodegenerative diseases, arthritis, and orthopedic conditions that affected upper extremity movement. See Table 1 for subject characteristics.

Clinicians: Inclusion criteria: Licensed Physical and Occupational Therapists with at least three years of experience treating persons with stroke.

#### 2.1.2. Data Collection

**Figure 1:**
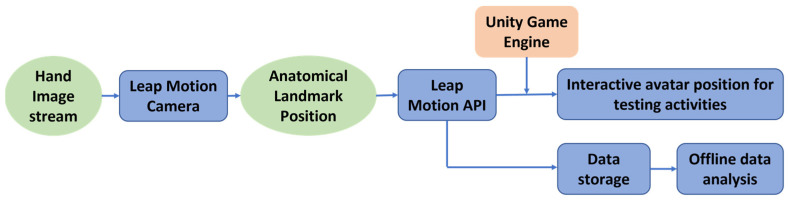
Data Collection Process.

NJIT-HoVRS utilizes the LMC to collect joint position data, which is used to control a library of games and collect kinematic testing data. The LMC is a combination of two cameras and three infrared LEDs. The cameras collect data in an apex-down conical field that begins approximately 10 cm above the device and ends approximately 60 cm above. The LMC’s cameras collect an image of the hand 120 times per second. Image data is streamed to the LMC’s USB controller, which reads anatomical landmark position data directly into its local memory and adjusts resolution as necessary. Data is streamed to the Leap Motion Image Application Programming Interface (API) via USB. We programmed the HoVRS system using Unity to feed anatomical landmark position data, which produces avatar movement during testing activities by calling the Leap Motion API. The same anatomical landmark position data is stored for offline analysis (see Figure 1). 

#### 2.1.3. Testing Set Up

**Figure 2:**
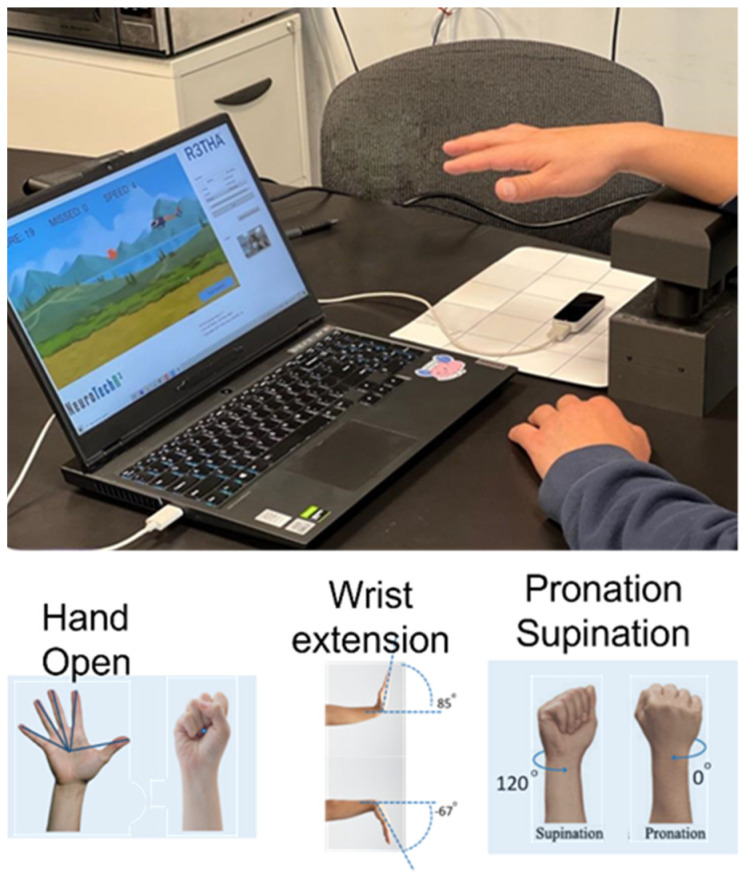
Orientation of camera and measurements (**Top**) Experimental set up; (**Bottom**) Movements measured by LMC.

The tests were performed with subjects seated at a table with their forearms resting on a 20 cm high arm support. We position the patient with their forearm supported 20 cm above the camera in order to give the subject ample room to flex their wrists with fingers extended below the arm support without the fingers getting too close to the camera/below its field of view. The front edge of the support was one centimeter proximal to the radial and ulnar styloid processes. LMC was positioned parallel to the arm support base, directly below the third metacarpophalengeal joint of the subjects’ hand. All measurements were collected in person, either in our laboratory or in our subjects’ homes (See Figure 2).

#### 2.1.4. Kinematic Measurements

**Figure 3:**
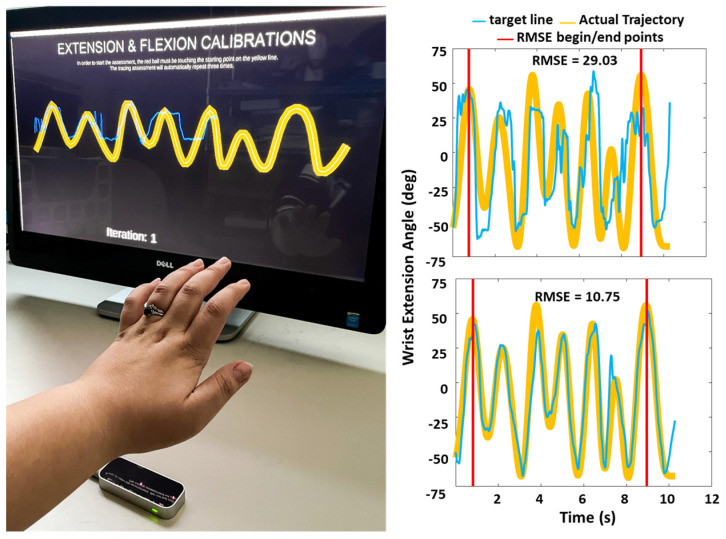
Accuracy Measures. **Left**: Photograph of subject performing Hand Opening Accuracy test. On the screen the thick yellow line is the target wave and bright blue line is generated by cursor, which is controlled by the subject. Subject is cued to keep the bright blue line within the yellow line. Note: Leap Motion Controller and table top arm support are positioned to optimize this photograph. Please see methods for actual positioning. **Right**: Calculation of accuracy measures. Examples of two representative performances and results for the WEA measure. Vertical red lines depict beginning and ending of data collection for Root Mean Square Error calculation. Yellow lines represent target line. Blue lines represent line generated by cursor controlled by subject flexing and extending their wrist. Scores for these two performances are in each panel.

(1) Hand Opening Range (HOR): The subjects opened their hands as much as possible and closed them as tightly as possible with their palms down. Subjects held each position for 3 s. The HOR value was calculated as the average distance of all five fingertips from a point on the volar surface of the wrist between the radial and ulnar styloid processes (see Figure 2).
(2) Hand Opening Accuracy (HOA): The subject controlled a computer cursor that moved up and down by opening and closing their hand. The subject traced an irregular wave (see Figure 3) that moved across the screen with the cursor at a rate of 2.5 cm per second. The highest point on the tracing wave required 80% of the maximum hand opening distance, and the lowest point required 80% of the maximum closing distance, as measured above. Subjects were cued to trace the wave with the cursor as closely as possible. The task was initiated by the subject moving the cursor to the stationary wave until the cursor met the first point on the wave. When this was achieved, the cursor moved across the screen without stopping until the trial was completed. Accuracy was calculated as the root mean square error between the cursor position and the corresponding target point on the wave. We began calculating accuracy after the cursor passed the first peak and stopped collecting after the cursor passed the last peak (see Figure 3). The task was repeated three times, and we report the best root mean square error (RMSE) of the three trials.
(3) Wrist Extension Range (WER): The subject extended and flexed their wrist against gravity with their forearm resting on the arm support. Subjects held each position for 3 s. Subjects were cued to move at the wrist only, and the trial was repeated if the subject moved their forearm. The wrist extension angle is calculated as the angle formed by the plane of the palm and the plane of the LMC (y-axis) (see Figure 2).
(4) Wrist Extension Accuracy (WEA): The subject controlled a cursor that moved up and down by flexing and extending their wrist. Wave amplitude and accuracy calculations were identical to HOA.
(5) Pronation-Supination Range (PSR): The subject pronated and supinated their hand against gravity while keeping their forearm resting on the arm support. Subjects were cued to move only at the forearm, and the trial was repeated if the subject moved their humerus or trunk. The pronation angle is calculated as the angle formed by the plane of the palm and the plane of the LMC around the z-axis (see Figure 2).
(6) Pronation-Supination Accuracy (PSA): The subject controlled a cursor that moved up and down by pronating and supinating their hand. The wave amplitude and accuracy calculation were identical to HOA.

Subjects are advised to re-do a test if they failed to perform a repetition according to the specifications above. If subjects were unable to perform a movement to the specifications above, they did not receive a score for that item. All analyses are performed without a score for that subject or that movement.

#### 2.1.5. System Usability Scale Testing

See Appendix A for SUS questions. There are ten items that the participant scores from 1 (strongly disagree) to 5 (strongly agree). Using the participant’s score, item scores are calculated as follows: For items 1, 3, 5, 7, and 9, the item score is the participant’s score minus 1. For items 2, 4, 6, 8, and 10, the item score is 5 minus the participant’s score. All ten items’ score contributions are summed and multiplied by 2.5 to achieve the composite score, ranging from 0 to 100 [24]. Higher scores are commensurate with higher levels of perceived usability [24]. In the field of human-computer interaction, it is widely accepted that a score of 68 or more on the SUS means that a device is “acceptable to use” [25].

#### 2.1.6. Testing Paradigms

Intra-rater reliability: We examined scores from a battery of six kinematic measurement scores (see below) for six persons with chronic stroke collected by six different licensed therapists three times during the same day to examine the test–retest reliability of the test battery and examine the impact of remote data collection on test scores. Prior to therapist and person with stroke interaction, therapists were trained to use the system launcher, which allows video conferencing with the person with stroke, to calibrate the system, and to perform kinematic assessments. All testing for this study was performed in our laboratory. During the first therapist/patient session (in-person session/Test 1), the therapist and person with stroke were in the same room, which allowed the therapist to teach the person with stroke how to set up the physical system, which includes a laptop, a LMC, an arm support, and a video conferencing camera (see Figure 2).

The therapists controlled the person with stroke’s system launcher via a remote desktop application. They then guided the person with a stroke through the six remote kinematic assessments (see Section 2.1.4 above). Subjects rested approximately twenty minutes between sessions. During the two subsequent sessions (Remote Session 1, and Remote Session 2/Test 2 & 3 respectively), the therapist and person with stroke were in separate rooms to simulate performing the tests remotely. Subjects set up the system in a separate room, without assistance or guidance from study staff. They communicated via video conferencing software, allowing the therapist and person with stroke to see each other. The therapist could also see the person with a stroke’s hand and arm during the remote sessions to ensure that they performed the tests correctly. They repeated the same steps for the kinematic assessments as the in-person session. 

After completing all three test sessions, the therapist and person with stroke completed a System Usability Survey [24].

#### 2.1.7. Statistical Analyses

All statistical analyses were performed in Minitab^®^ 20.4. The normality of each data set was confirmed using the Kolmogorov–Smirnov test. When subjects were unable to perform a specific task, the corresponding analyses for that task were calculated without their data. See the n for each analysis in Table 2.

#### 2.1.8. Intra-Rater Reliability

The three sets of kinematic test scores collected on the same day were evaluated using two-way, random effects, single rater consistency intra-class correlation coefficients (ICC(2,1)). Values less than 0.5 are indicative of poor reliability; values between 0.5 and 0.75 indicate moderate reliability; values between 0.75 and 0.9 indicate good reliability; and values greater than 0.90 indicate excellent reliability. We compared the first in-person session score and the remote Session 1 score to evaluate the agreement between the scores collected in person and those collected remotely. We compared the remote Session 1 and remote Session 2 scores to evaluate the agreement between repeated collections of scores in the remote condition.

#### 2.1.9. System Usability Scale

SUS Scores were calculated using methods described by Brooke [24]. The ten statements on the survey alternate between positive and negative, therefore each item’s contribution (between 0–4) must be calculated. SUS scores for both groups were evaluated for normality. Means and standard deviations are reported.

### 2.2. Results

#### 2.2.1. Subjects

There were seven subjects in this study. See Table 1 for subject demographics.

#### 2.2.2. Intra-Rater Reliability Study

When comparing the in-person session (Test 1) to the remote session 1 (Test 2), the ICC for four of the six measurements (HOA, WEA, PSR, and PSA) were excellent. The ICCs for WER and HOR were good. When comparing Remote Session 1 (Test 2) to Remote Session 2 (Test 3), the ICCs for four of the six measurements (WER, WEA, PSR, and PSA) were excellent. The ICC for HOR and HOA were good (see Table 2).

#### 2.2.3. System Usability Scale Scores

The therapist’s SUS scores ranged from 70 to 90. The mean was 83.1 (SD = 6.4), which is above the published cutoff score of 68 [25]. Person with stroke SUS scores ranged from 70 to 85. The mean was 80.4 (SD = 5.2).

## 3. Study #2

### 3.1. Materials and Methods

#### 3.1.1. Subjects

Subjects were recruited through stroke support group activities and two assistive technology fairs. Sixteen subjects participated in Study 2. Inclusion and exclusion criteria: See Study 1. All subjects were free from neurodegenerative diseases, arthritis, and orthopedic conditions that affected upper extremity movement.

#### 3.1.2. Data Collection, Testing Set Up and Measurements

See Study 1.

#### 3.1.3. Clinical Testing

We utilized the UEFMA as a measure of impairment caused by stroke, using the protocol described by Deakin et al. [26]. The test was performed by a Licensed Physical Therapist with extensive experience working with persons with stroke. All measurements were collected in person, either in our laboratory or in our subjects’ homes. The UEFMA produces scores between 0 and 66. Higher scores are commensurate with higher levels of recovery from stroke.

#### 3.1.4. Testing Paradigms

We performed two sets of tests to examine the ability of our testing battery to identify impaired movement.

Discrimination test: First, we examined the same battery of kinematic measurements as in Study # 1 in sixteen persons with chronic stroke to determine if these scores could distinguish the performance of the subjects’ paretic hands from their non-paretic hands. For this study, subjects performed the six tests in order, from beginning to end, one time with their non-paretic hand. After this, they performed testing activities in an identical fashion with their paretic hand. We tested the scores from these two samples to see if the test battery could distinguish normal hand function from paretic hand function in individual subjects.

Convergent validity test: After kinematic testing, the same sixteen subjects performed the UEFMA. We examined correlations between individual kinematic scores and the UEFMA score and the difference between an impaired and unimpaired hand score to examine the relationship between the kinematic test battery scores and overall UE impairment. 

#### 3.1.5. Statistical Analyses

All statistical analyses were performed in Minitab^®^ 20.4. The normality of each data set was confirmed using the Kolmogorov–Smirnov test. When subjects were unable to perform a specific task, the corresponding analyses for that task were calculated without their data. 

#### 3.1.6. Discriminatory Analyses

Kinematic scores collected for each of the six measurements with stroke subjects’ unimpaired hands were compared to scores collected with their impaired hands and evaluated with paired, two tailed *t*-tests. 

#### 3.1.7. Correlational Analyses

Two sets of correlations examining the relationship between our six kinematic measurements provided during remote testing and the level of impairment as measured by the UEFMA score were evaluated using Pearson Correlation Coefficients (PCC). First, the correlation between kinematic scores and UEFMA of the subjects’ impaired hands was evaluated. In addition, difference scores for each kinematic measure (impaired hand score minus unimpaired hand score) were calculated, and the correlation between the difference score and the UEFMA was evaluated. PCCs of 0.100 to 0.399 are considered weak. PCC values between 0.400 and 0.699 are considered moderate.

### 3.2. Results

#### 3.2.1. Subjects

There were 16 subjects in this study. See Table 3 for subject demographics.

#### 3.2.2. Discriminatory Analyses

1 subject was unable to perform the WEA task. Two subjects were unable to perform the PSR or PSA tasks. There were statistically significant differences in the kinematic scores when comparing unimpaired and impaired UE for all six measures in subjects with stroke (See Table 4). 

#### 3.2.3. Correlations between Impaired Hand Scores and UEFMA

**Figure 4:**
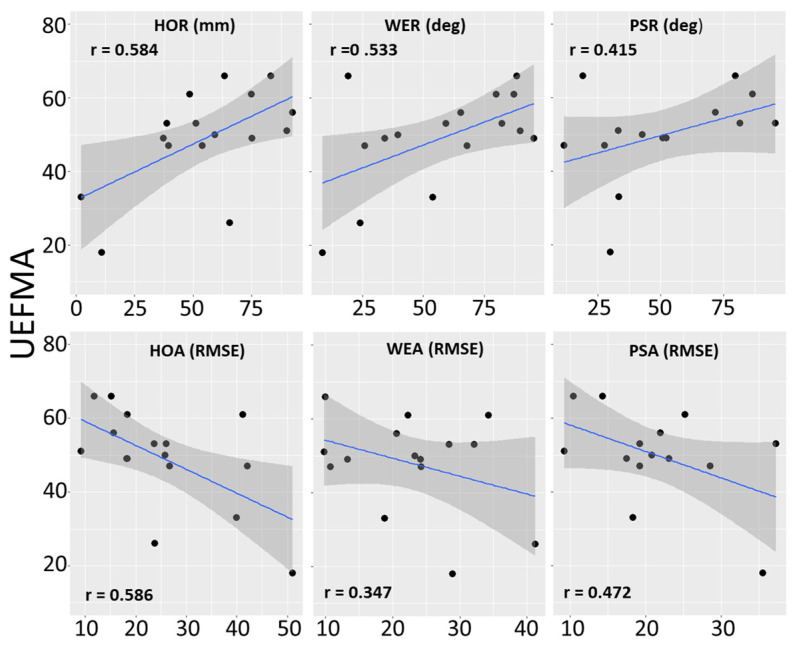
Correlations between LMC measures and Upper Extremity Fugl-Meyer Assessment.

The correlations between the impaired hand scores for HOR, HOA, and WER and the UEFMA score were moderate and statistically significant. The correlations between impaired hand scores for PSR and PSA were moderate but not statistically significant. (See Table 5 and Figure 4). 

The correlations between Upper Extremity Fugl-Meyer Assessment (UEFMA) scores and the six Leap Motion Controller collected measurements. All tests are performed in a laboratory setting. Shaded gray areas are 95% confidence intervals of the blue regression line. Larger range numbers on the top row (HOR, WER, and PSR) indicate better performance. Smaller RMSEs on the bottom row (HOA, WEA, and PSA) indicate better performance.

#### 3.2.4. Correlations between Difference Scores and UEFMA

**Figure 5:**
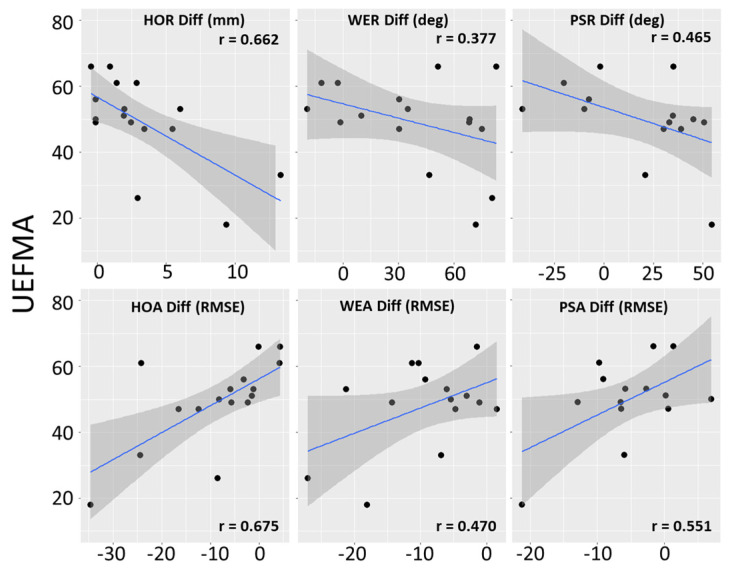
Correlations between Differences in Impaired and Unimpaired Hand LMC measures and Upper extremity Fugl-Meyer Assessment.

The correlations between the difference scores for HOR, HOA, and PSA and the UEFMA score were moderate and statistically significant. The correlations between the difference scores for WER and PSR with UEFMA were moderate but not statistically significant. (See Table 5 and Figure 5).

Correlations between Upper Extremity Fugl-Meyer Assessment (UEFMA) scores and differences between impaired and unimpaired hand scores for the six Leap Motion Controller collected measurements. All tests are performed in a laboratory setting. Shaded gray areas are 95% confidence intervals of the blue regression line. Small positive differences on the top row (HOR, WER) indicate that impaired upper extremity range is similar to unimpaired upper extremity range. Negative differences on the top row (PSR) indicate that the impaired upper extremity range is larger than the unimpaired upper extremity range. Negative numbers on the top row indicate that impaired upper extremity errors were larger than unimpaired upper extremities.

## 4. Discussion

This study examined the intra-rater reliability, usability, and validity of six camera-based measures of hand and wrist function in persons with stroke. The reliability and validity studies described in this paper produced mixed results, suggesting that further refinement is necessary, but that clinical utilization of these measures may be reasonable. Overall inter-rater reliability for 5 of the 6 measures was above 0.900, suggesting that they are acceptable for clinical practice [27,28,29,30].

Several authors question the accuracy of finger joint angle calculations produced by the LMC tracking software [31,32]. To bypass these issues, we utilize the average distance between the fingertips and palm to measure the ability to open and close the hand maximally and the ability to control hand opening and closing movement. This approach does not allow for tracking the changes in the excursions of individual joints but provides a rough measure of the ability to open and close the hand. The data presented in this study suggests that these measures of hand opening and closing may be reliable and valid. We also use this approach when developing training games that train hand opening and closing as well as individual finger movement [8].

All six NJIT—HoVRS based measures were able to distinguish the impaired from the unimpaired hands of subjects. Beyond serving as a minimum criterion for clinical utility, this method of validation was utilized in an attempt to reduce bias introduced by the use of a camera by roughly standardizing hand size within each subject [19]. We considered this analysis to be particularly important for our hand opening measurement, which is distance based. Five of the correlations between LMC-based kinematic measures using the impaired hand and UEFMA were moderate. Difference scores performed in a similar fashion, but interestingly, correlations between the difference scores were stronger than impaired hand scores for five out of six of the kinematic measures. We plan to continue evaluating these two approaches to scoring the kinematic tests.

SUS scores for persons with stroke and therapists were both above 80, which is comparable to an automated version of the Box and Blocks test in persons with stroke [33] and two LMC-based rehabilitation systems in persons with stroke [34,35]. In the field of human-computer interaction, it is widely accepted that a score of 68 or more on the SUS means that a device is “acceptable to use” [36]. However, this criterion does not necessarily translate into broad adoption in the field by clinicians [25]. More extensive implementation studies and system refinement will be necessary to maximize the translation of this approach to measurement into clinical practice.

The most prominent limitation of this study is our choice to test the system in a laboratory setting. Further testing in an actual home setting is necessary to validate the testing process as a telerehabilitation option. The agreement between simulated remote testing and same-room testing was excellent for four of the six measurements and good for the other two. In addition, subjects in our pilot study of NJIT—HoVRS were trained to productively use the system with no supervision from study personnel [9]. Taken together, this suggests the setup and execution of testing procedures using identical equipment and movements by the same population in their homes should be feasible and effective. Another limitation is the small number of subjects for the individual experiments. The data presented in this study will serve as pilot data for a larger study. Further validation of our kinematic testing will include responsiveness and expanding our convergent validation to activity level tests.

## 5. Conclusions

The set of six measurements presented in this paper demonstrate levels of intra-rater reliability that are consistent with clinical practice. The remote collection of these tests does not seem to have a substantial impact on scores. The tests could distinguish normal from impaired movement, and scores are moderately correlated with a clinical measure of impairment. These findings suggest that using these measurements in clinical practice might be reasonable, and further refinement and study are indicated.

## Figures and Tables

**Table 1:** Study #1 Subject Characteristics.

Subj	Age	Sex	Months Since CVA	Premorbid Handedness	Impaired Hand	UEFMA
1	51	M	8	L	R	66
2	44	F	12	R	R	56
3	53	M	7	L	L	66
4	74	M	47	R	L	53
5	29	M	36	L	L	51
6	49	M	53	R	L	15
7	60	F	37	R	L	44

**Table 2:** Agreement between scores collected across testing sessions.

Measurement	*n*	ICC Test 1–2	Test 1–2 95% CI	ICC Test 2–3	Test 2–3 95% CI
HOR	7	0.857	0.381–0.973	0.824	0.280–0.967
HOA	6	0.910	0.496–0.987	0.794	0.098–0.968
WER	7	0.883	0.472–0.979	0.905	0.549–0.983
WEA	6	0.581	(-) 0.309–0.928	0.676	(-) 0.161–0.947
PSR	7	0.911	0.577–0.984	0.967	0.820–0.994
PSA	7	0.969	0.831–0.995	0.785	0.176–0.959

CI = Confidence Interval.

**Table 3:** Study #2 Subject Characteristics.

Subj	Age	Sex	Time Since CVA (Months)	Premorbid Handedness	Impaired Hand	UEFMA
1	51	M	8	L	R	66
2	44	F	12	R	R	56
3	53	M	7	L	L	66
4	52	F	14	R	L	56
5	63	M	8	R	R	20
6	86	M	6	R	R	55
7	68	M	264	R	L	50
8	49	M	53	R	L	15
9	57	M	6	R	L	30
10	59	M	6	R	R	49
11	76	F	9	R	R	49
12	67	M	161	R	R	61
13	60	M	6	R	L	53
14	74	M	47	R	L	53
15	29	M	36	L	L	51
16	64	M	18	R	L	26

**Table 4:** Comparisons between Unimpaired and Impaired Upper Extremity Scores.

Measurement	*n*	Unimpaired	Impaired	Mean Diff	95% CI	T	*p*
HOR (mm)	16	88(21)	53(27)	55 (37)	36.8–73.1	3.41	0.002 *
HOA (RMSE)	16	17(6)	25(12)	25.4 (11.1)	19.6–30.3	(-) 3.16	0.006 *
WER (deg)	16	97(18)	57(29)	57.5 (34.9)	40.3–74.6	4.42	<0.001 *
WEA (RMSE)	15	14(5)	23(9.5)	22.8 (8.0)	18.8–26.8	(-) 4.44	<0.001 *
PSR (deg)	14	68(13)	51(28)	51.6 (29.4)	36.2–67.0	2.37	0.016 *
PSA (RMSE)	14	17(8)	21(9)	21.5 (7.0)	17.9–25.1	(-) 2.7	0.015 *

Correlational analyses: 1 subject was unable to perform the WEA task. Two subjects were unable to perform the PSR or PSA tasks. CI = Confidence Interval. * = statistically significant comparison.

**Table 5:** Correlations between Kinematic Scores and Upper Extremity Fugl-Meyer Assessment Scores.

Measure	*n*	Impaired: UEFMA	*p*	Impaired–Unimpaired Difference: UEFMA	*p*
HOR	16	0.584	0.017 *	0.662	0.005 *
HOA	16	0.586	0.017 *	0.675	0.004 *
WER	16	0.533	0.033 *	0.377	0.150
WEA	15	0.347	0.205	0.470	0.077
PSR	15	0.415	0.124	0.465	0.081
PSA	14	0.472	0.088	0.551	0.041 *

* = <0.05.

## Data Availability

De-identified data will be supplied by the corresponding author if requested in writing.

## Appendix A

System Usability Scale Questions

1. I think that I would like to use this system frequently.
2. I found the system unnecessarily complex.
3. I thought the system was easy to use.
4. I think that I would need the support of a technical person to be able to use this system.
5. I found the various functions in this system were well integrated.
6. I thought there was too much inconsistency in this system.
7. I would imagine that most people would learn to use this system very quickly.
8. I found the system very cumbersome to use.
9. I felt very confident using the system.
10. I needed to learn a lot of things before I could get going with this system.
